# An updated overview on the bacterial PhoP/PhoQ two-component signal transduction system

**DOI:** 10.3389/fcimb.2025.1509037

**Published:** 2025-01-31

**Authors:** Meiqin Mao, Li He, Qingpi Yan

**Affiliations:** Fisheries College, Jimei University, Xiamen, Fujian, China

**Keywords:** bacterial, TCS, PhoP/PhoQ, phosphorylation, virulence

## Abstract

The PhoP response regulator and the cognate sensor kinase PhoQ form one of the two-component signal transduction systems that is highly conserved in bacteria. The PhoP/PhoQ system is a crucial mediator of signal transduction. It regulates the expression of bacterial environmental tolerance genes, virulence factors, adhesion, and invasion-related genes by sensing various environmental signals in the host, including Mg^2+^, low pH, antimicrobial peptides, and osmotic pressure. In this review, we describe the PhoP/PhoQ system-induced signal composition and its feedback mechanism, and the abundance of PhoP phosphorylation in the activated state directly or indirectly controls the transcription and expression of related genes, regulating bacterial stability. Then, we discuss the relationship between the PhoP/PhoQ system and other components of the TCS system. Under the same induction conditions, their interaction relationship determines whether bacteria can quickly restore their homeostasis and exert virulence effects. Finally, we investigate the coordinated role of the PhoP/PhoQ system in acquiring pathogenic virulence.

## Introduction

**Figure 1:**
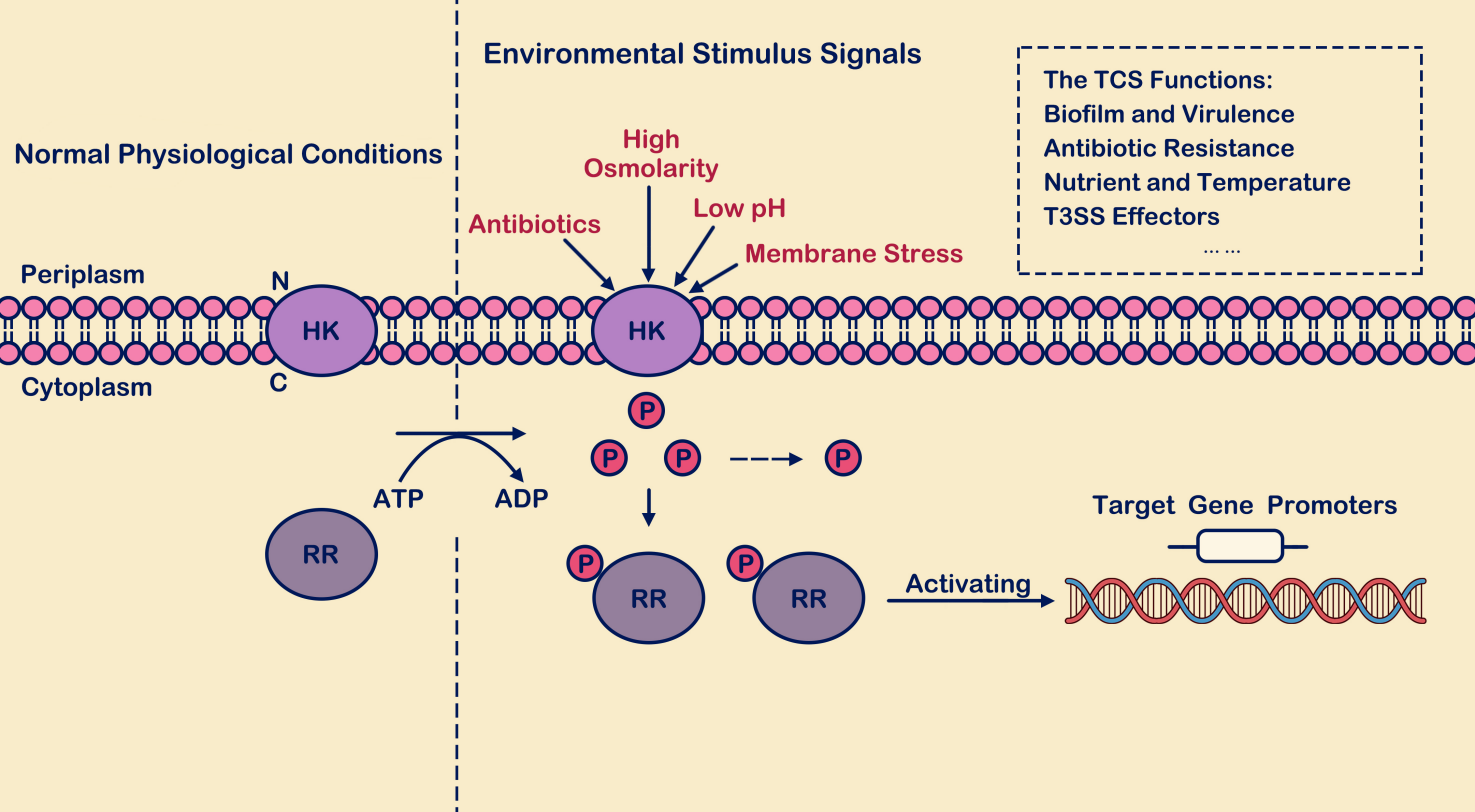
The process of two-component signal transduction. Under environmental stimulus conditions, histidine kinase (HK) interacts with its signaling ligand, leading to phosphorylation of histidine residue. The phosphate group is recognized and captured by response regulator (RR) in the cytoplasm, activating the output domains of the response regulator and inducing conformational changes. Subsequently, the regulator binds to the promoter regions of downstream target genes, thereby activating transcription levels of both themselves and downstream target genes. Here, “P” denotes the phosphate group, “HK” stands for histidine kinase, and “RR” represents response regulator.

Bacteria may encounter various environmental pressures, affecting their survival and virulence (Yao et al., 2024; He et al., 2025). In response to environmental pressure, many strategies have been evolved to fight against external pressure. The two-component signal transduction systems (TCSs) play an essential role in signal transduction during the change of bacterial environment (Xie et al., 2022). It enables bacterial pathogens to sense various environmental conditions such as light, temperature, pH, osmotic pressure, nutrients, small molecule metabolites, antibiotics, antimicrobial peptides, and other host-derived signals. This ability allows pathogens to determine when they have reached the microenvironment of a host or host interior. Subsequently, specific genes are activated or repressed to adapt, evade, or attack (Xie et al., 2022). The two-component signal transduction systems consist of conserved signal receivers: histidine kinases (HKs) and their cognate response regulators (RRs) (Xie et al., 2020). Studies have shown that TCS usually uses positive and negative feedback mechanisms to regulate gene expression in HK, RR, and downstream genes (**Figure 1**). In this way, PhoP/PhoQ system responds positively to environmental stress (Lippa et al., 2009). Depending on the structural domain is divided into six families, respectively: the OmpR family, the family of NarL, the NtrC family, the LuxR family, the CitB family, and Che (Pina et al., 2021), TCSs control the various components of the phosphate transferring principle almost similar; they form between complex signal transportation network (Véscovi et al., 1996; Pina et al., 2021; Shao et al., 2021). The composition of bacterial TCS is rich, forming a complex information transportation network between them. However, the TCS system has not been found in animal hosts, and as a signal transduction system, TCS could be a new target for developing new antibacterial therapeutic agents (Chen and Groisman, 2013).

The PhoP/PhoQ system, a member of the OmpR family, has been thoroughly studied in *Salmonella enterica* (*S. enterica*), *Escherichia coli* (*E. coli*), *Shigella flexneri* and other bacterial strains (Lin et al., 2017; Yuan et al., 2017; Pina et al., 2021; Guo et al., 2022), and plays an important role in the entire regulatory network (Pina et al., 2021). In *S. enterica*, PhoP/PhoQ is involved in regulating the transcription and expression of a variety of virulence genes, including invasion of non-phagocytic cells (such as epithelial cells), anti-phagosome killing, resistance to antimicrobial peptides (AMPs), and release of virulence proteins (Pina et al., 2021). The PhoP/PhoQ system consists of two parts: PhoQ belongs to transmembrane proteins, and its structural composition mainly includes the N-terminal conserved periplasmic sensor domain, two transmembrane (TM) domains, the histidine adenosine monophosphate associated protein (HAMP) domain located in the cytosol for signal transmission, the dimerization and histidine phosphotransfer (DHp) domain required for dimerization, and the catalytic adenosine (CA) domain that binds to catalytic adenosine triphosphate (ATP) (Mensa et al., 2021). PhoP, a homologous regulatory factor (RRs) located downstream of PhoQ, consists of two domains: the N-terminal regulatory domain, which has the necessary aspartate residue site, and one is the C-terminal effect domain, which is involved in binding to the specific DNA sequence in the target promoter (Ali and Abdel Aziz, 2024). The catalytic and regulatory structures of these two proteins are relatively conserved. PhoQ is commonly used as a sensor to recognize environmental stimuli, promote self-phosphorylation of histidine residues under the catalysis of ATP, and deliver phosphate groups to its cognate regulator PhoP (Yamamoto et al., 2002; Pathak et al., 2010). After the N-terminal aspartic acid residue of PhoP is captured and recognized, the phosphorylation reaction (PhoP-P) occurs, and the PhoP conformation changes (Yamamoto et al., 2002; Pathak et al., 2010). PhoQ controls PhoP phosphorylation and influences the transcription of PhoP-regulating genes (Gall et al., 2016; Mattos-Graner and Duncan, 2017). Following phosphorylation of PhoP, on the one hand, it can promote its own transcription and activate the expression of downstream gene targets (such as *mgtA*, *slyB*, *pmrD*, *pagP*). More so, it can competitively bind with other transcription factors, resulting in down-regulation of specific gene targets (Goldberg et al., 2010). The activated PhoP/PhoQ system mediates various phenotypic modifications, regulates bacterial homeostasis, and reduces the adverse effects of external environmental pressure (Goldberg et al., 2010).

## How does the PhoP/PhoQ system respond to external environmental stimulus signals?

The PhoP/PhoQ system, as a classic two-component system, involves the dual-function protein PhoQ, which senses environmental changes such as divalent cations (Véscovi et al., 1996), antibacterial (Yu and Guo, 2011), low pH (Bader et al., 2010), circumcellular redox (Choi and Groisman, 2016), and osmotic pressure (Yuan et al., 2017). These factors regulate the phosphorylation-mediated phenotypic modification of the response regulator PhoP. The PhoP/PhoQ system plays a crucial regulatory role in virulence of in the virulence of several pathogenic bacteria. Therefore, elucidating the response mechanisms of the PhoP/PhoQ system to various stimuli and its transcriptional regulation of downstream target genes provides fundamental insights into the PhoP/PhoQ system.

### Divalent cations

Divalent cations play a crucial role in organisms, serving as essential cofactors for numerous enzymes. They are vital for maintaining the integrity of biological membranes and facilitating various physiological functions (Lippa and Goulian, 2012). Mg^2+^ was initially identified as the environmental stimulus factor for the PhoP/PhoQ system, which plays a crucial role in maintaining Mg^2+^ homeostasis (Véscovi et al., 1996). When the cytoplasmic Mg^2+^ concentration falls below a certain threshold (e.g., when *Salmonella typhimurium* concentration below 0.5 mM Mg^2+^) (Véscovi et al., 1996), bacteria generally reduce the assembly of functional ribosomes and undergo auto-phosphorylation of the periplasmic PhoQ. PhoP is phosphorylated to PhoP-P, and PhoP-P specifically binds to the promoter region of Mg^2+^ transport-related genes (such as *mgtA*, *mgtB*, and *mgtC*), and thus activating gene transcription (Cromie and Groisman, 2010; Yeom et al., 2020; Yeom and Groisman, 2021). In the case of *E. coli*, when Mg^2+^ levels decrease to levels impairing protein production (below 10 μM Mg^2+^), PhoP-P promotes the expression of the *iraP* gene. This increases the intracellular content of RpoS, reducing the rate of protein synthesis to maintain essential cellular functions (Yin et al., 2019). Meanwhile, the expression level of the Mg^2+^ transporter protein MgtA is significantly upregulated, facilitating the transport of Mg^2+^ from the periplasm to the cytoplasm, thereby maintaining stable cellular Mg^2+^ concentrations (Park et al., 2018). When PhoQ is activated by cationic antimicrobial peptides or acidic environmental conditions, MgtA remains unaffected (Subramani et al., 2016).

Due to environmental stress, the PhoP/PhoQ cascade activates the transcription of downstream genes, which requires significant ATP consumption. The availability of ATP directly correlates with changes in the abundance of ClpXP (Groisman, 2016). Under normal conditions, upon binding with adaptor proteins, RpoS is transported to ClpXP for degradation, rapidly reducing RpoS levels (Groisman, 2016). The transcription factor RpoS regulates the expression of numerous bacterial genes, with its synthesis and degradation tightly controlled, varying in response to cellular growth stresses (Battesti and Gottesman, 2013; Schellhorn, 2020). In *Salmonella* enterica serovar Typhimurium (*S. Typhimurium*) under low Mg^2+^ conditions (≤20 μM Mg^2+^), the stability of the sigma factor RpoS plays a crucial role in the PhoP/PhoQ system cascade (Bougdour et al., 2008). PhoP-P promotes the upregulation of RssB anti-adaptors (IraM/IraP/IraD) expression (Bougdour et al., 2008). Acting as an intermediary in regulating RpoS stability, it interferes with RssB-mediated degradation of RpoS by interacting with RssB. Moreover, the PhoP/PhoQ cascade promotes the regulation of RpoS stability by *iraP*, and high levels of RpoS mediate transcriptional expression of its dependent genes (such as *katE* and *esrB* genes) (Bougdour et al., 2008).

SlyB, located in the outer membrane, is regulated by PhoP-P under decreased Mg^2+^ concentration (such as in *Yersinia pestis* when Mg^2+^ is below 50 μM) or increased osmotic pressure (such as in *E. coli* when stimulated with 300 mM NaCl) (Tu et al., 2006; Perez et al., 2009; Yuan et al., 2017). In addition, SlyB plays a negative regulatory role in some bacteria on PhoP/PhoQ (Tu et al., 2006; Perez et al., 2009). For instance, in *Salmonella typhimurium*, deletion of the *slyB* gene leads to decreased transcription levels of genes activated by PhoP-P. In contrast, such a negative regulatory mechanism is not observed in *E. coli* (Lippa et al., 2009). Additionally, SlyB can respond to outer membrane (OM) biogenesis defects by sensing the accumulation of lipopolysaccharide (LPS) and periplasmic unfolded outer membrane proteins (OMPs). The modification of LPS plays a crucial role in the PhoP/PhoQ cascade (Janssens et al., 2024). LPS modifications help bacteria reduce the electrostatic repulsion of phosphorylated residues and releases a certain amount of Mg^2+^ for MgtA-related proteins to transfer Mg^2+^ from the periplasmic space into the cytoplasm (Janssens et al., 2024). Studies on *S. Typhimurium* demonstrate that under low Mg^2+^ conditions (less than 50 μM Mg^2+^), the *mgtA* gene is activated in a PhoP-P-dependent manner, independent of other environmental stimuli (Yeom et al., 2020). When PhoQ detects signals like low pH or antimicrobial peptides, the expression level of the Mg^2+^ transporter gene *mgtA* remains unaffected (Yeom et al., 2020; Groisman et al., 2021). Additionally, Ca^2+^ and Mn^2+^ can serve as ligands for PhoQ with similar mechanisms of action, neutralizing electrostatic repulsion between negatively charged residues at the divalent cation binding sites (Regelmann et al., 2002; Barchiesi et al., 2008). Conversely, as the concentration of divalent cations increases, the expression levels of regulatory proteins produced by the PhoP/PhoQ cascade (such as PgtE, PhoN, MgtA, MgtB, and IraP) gradually decrease (Cho et al., 2006; Bougdour et al., 2008). When the Mg^2+^ concentration exceeds 50 μM, a stable bridge forms between the negatively charged outer and inner membranes, thereby inhibiting the PhoP/PhoQ cascade reaction (Regelmann et al., 2002).

**Figure 2:**
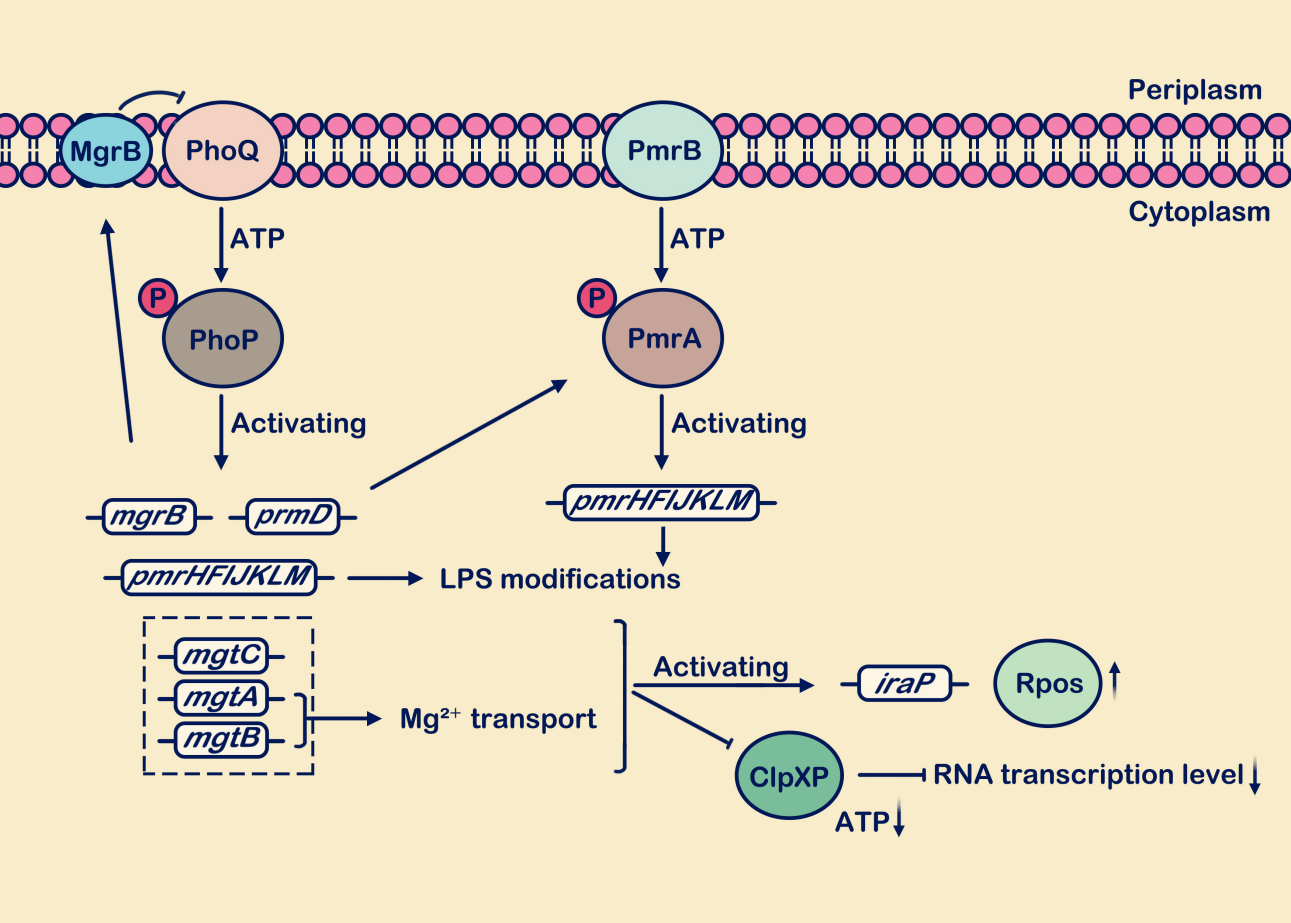
Low Mg^2+^ levels stimulate the activation of the PhoP/PhoQ and PmrA/PmrB systems. During growth under low Mg^2+^ conditions, the PhoP/PhoQ system induces transcription of target genes including *mgrB*, *pmrD*, *pmrHFIJKLM*, *mgtC*, *mgtA*, and *mgtB*. The *mgrB* gene is transcriptionally upregulated, and the synthesized MgrB membrane protein exerts negative feedback on PhoQ. Activation of the *pmrD* gene positively regulates the PmrA/PmrB system. The *mgtA* and *mgtB* genes facilitate the transport of extracellular Mg^2+^ into the cell. Presence of the *mgtC*, *mgtA*, and *mgtB* genes reduces ATP consumption and decreases protein synthesis rates. Activation of *pmrHFIJKLM* is involved in LPS modification. In the figure legend, a circle with “P” denotes a phosphate group, an upward vertical arrow indicates upregulation, a downward vertical arrow indicates downregulation, and the arrow from MgrB to PhoQ signifies “inhibition”.

Under conditions of low Mg^2+^ concentration (such as *S. Typhimurium* in a minimal medium containing 10 μM Mg^2+^), the PhoP/PhoQ system interacts with PmrA/PmrB (Hu et al., 2016). PmrA serves as the sensor responding to external stimulus signals, while PmrB acts as the downstream responder to PmrA (Kato et al., 2003; Paredes et al., 2023). PhoP-P stimulates the transcription of PmrD, which mediates the phosphorylation of another response regulator, PmrA. Sufficient PmrA-P is produced to promote the expression levels of genes such as *pmrC*, *pmrE*, *pmrHFIJKLM*, collectively modifying the outer membrane LPS (Chen and Groisman, 2013; Shprung et al., 2021; Paredes et al., 2023; Janssens et al., 2024). In *pmrD* deletion strains, it was found that the expression level of PmrA was significantly reduced compared to wild-type strains (Cho et al., 2006). Additionally, under high Fe^3+^ conditions, (such as *S. enterica* in a minimal medium containing 100 μM Fe^3+^) activate the PmrA/PmrB system (Bolard et al., 2019). PmrD also plays a role in promoting the activation of PmrA (Cho et al., 2006), serving as a crucial bridge between the PhoP/PhoQ and PmrA/PmrB systems, directly influencing the regulatory mechanism and abundance of PmrA (Cho et al., 2006). The above content is briefly described in **Figure 2**.

### Antimicrobial peptides

Antimicrobial peptides are widely sourced from diverse origins, including animals, plants, microorganisms, and synthetic production, serving as integral components of the innate immune systems in most multicellular organisms (Kato et al., 2012). AMPs are predominantly concentrated within phagosomes, where they exert antimicrobial effects in macrophages (Li et al., 2022). AMPs are rich in positive charges, enabling them to bind with negatively charged molecules on bacterial surfaces (Yan et al., 2021; Zhu et al., 2022). They swiftly penetrate lipid membranes, forming pores in bacterial cell membranes and disrupting membrane permeability, ultimately causing bacterial cell lysis (Yan et al., 2021; Zhu et al., 2022). For pathogens, resistance to antimicrobial peptides is crucial for exerting their toxicity. It has been established that antimicrobial peptides serve as direct signals for activating the PhoQ histidine kinase (Ramezanifard et al., 2023). Cationic antimicrobial peptides competitively bind to the periplasmic domain of PhoQ with divalent cations, inducing a conformational change in the cytoplasmic dimer (Ramezanifard et al., 2023). This promotes the phosphorylation of PhoP and alters the total charge of the lipid A portion of bacterial lipopolysaccharide (LPS), modifying LPS to increase bacterial resistance (Bader et al., 2005; Yu and Guo, 2011; Ramezanifard et al., 2023). The inner membrane protein Mig-14 in extraintestinal pathogenic *E. coli* (ExPEC) and *S. typhimurium*) play a crucial role within macrophages, significantly enhancing bacterial resistance against AMPs (Zhuge et al., 2018; Martynowycz et al., 2019).

**Figure 3:**
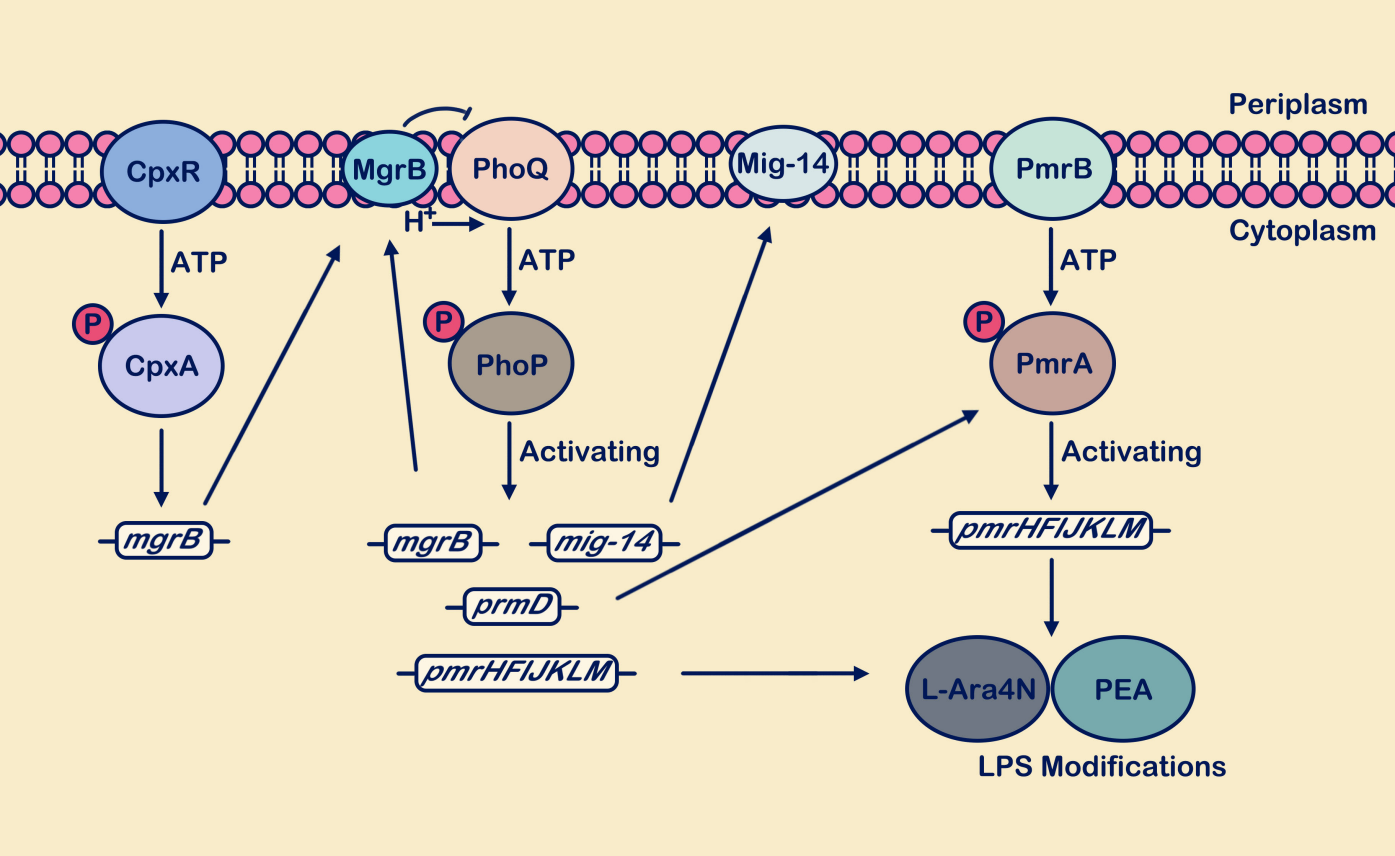
The PhoP/PhoQ and PmrA/PmrB systems synergistically respond to antimicrobial peptide attacks. Activation of the PhoP/PhoQ system promotes transcription of *mgrB*, *mig-14*, *pmrD*, and *pmrHFIJKLM*. The *mgrB* gene is upregulated, and the synthesized MgrB membrane protein exerts negative feedback on PhoQ. Upregulation of the *mig-14* gene supports the synthesis of the inner membrane protein Mig-14. Activation of the *pmrD* gene positively regulates the PmrA/PmrB system. Activation of *pmrHFIJKLM* facilitates the synthesis of L-Ara4N and PEA, which are used for LPS site modification, enhancing bacterial resistance. In the figure legend, a circle with “P” denotes a phosphate group, and the arrow from MgrB to PhoQ signifies “inhibition”.

In recent years, polymyxins have garnered significant attention from researchers due to the rapid increase in bacterial antibiotic resistance (Brodsky et al., 2005; Wang et al., 2020). Polymyxins are important cyclic peptide antibiotics isolated from Bacillus species (Gahlot et al., 2024). They disrupt membrane integrity and induce bacterial outer membrane damage by interacting with negatively charged surface structures such as LPSs in Gram-negative bacteria and lipoteichoic acids in Gram-positive bacteria, thereby exhibiting bactericidal activity (Storm et al., 1977; Brodsky et al., 2005). The cascade of PhoP/PhoQ system modifying bacterial outer membrane LPS can lead to increased resistance of bacteria to polymyxins (Guo et al., 2022). Meanwhile, PhoP-P regulates the expression of PmrD, which effectively inhibits the dephosphorylation of PmrA-P, thereby mediating the involvement of the PmrA/PmrB system in the modification process of LPS (Brodsky et al., 2005; Zhang et al., 2022). PhoQ promotes the binding of PhoP-P to its downstream *pmrHFIJKLM* promoter (also known as *arnBCADTEF* or *pbgPE* operator) through the recognition of polymyxin (Chandler et al., 2020). Concurrently, the upregulation of PmrD expression indirectly enhances the cascade reaction of the PmrA/PmrB system. PmrA also binds to downstream *pmrC*, *pmrE*, and *pmrHFIJKLM* promoters (Shprung et al., 2012; Poirel et al., 2017; Paredes et al., 2023). The overexpression products of *pmrC*, *pmrE*, and *pmrHFIJKLM* are utilized for the synthesis of 4-amino-4-deoxy-L-arabinose (L-Ara4N) and phosphoethanolamine (PEA) (**Figure 3**). These two components modify LPS by increasing the negative charge on the outer membrane (Hiroshi, 2003), reducing membrane permeability, thereby limiting the entry of antimicrobial peptides and playing a crucial role in promoting polymyxin resistance (Phan et al., 2017; Liu et al., 2021; Shahzad et al., 2023).

Typically, the regulation of its own and downstream target genes by the PhoP/PhoQ system to resist external stimuli is called positive feedback regulation. Negative feedback regulatory mechanisms collectively contribute in maintaining cellular homeostasis, and adverse feedback effects also play a crucial role in reducing intra-population cellular variability (Lippa et al., 2009). The membrane protein MgrB activation occurs through PhoP phosphorylation (Lippa et al., 2009). Subsequently, it binds to the periplasmic domain of PhoQ in order to attenuate its interaction with other stimulus signals, thereby inhibiting the phosphorylation of PhoP and establishing a negative feedback mechanism (Lippa et al., 2009; Poirel et al., 2017). The combination of positive and negative feedback in the PhoP/PhoQ system enhances bacterial sensitivity to signals and plays a crucial role in maintaining intracellular homeostasis (Lippa et al., 2009). When MgrB undergoes functional changes or is lost, the negative feedback regulation of PhoPQ is disrupted (Zafer et al., 2019; Kong et al., 2021). Although the absence of MgrB indirectly affects the activation of PmrD, MgrB appears to specifically target the PhoQ domain (Zafer et al., 2019). In PhoQ-deficient strains, MgrB does not exert its inhibitory effect and does not influence PmrD-mediated resistance to polymyxin B (Zafer et al., 2019). Recent studies have suggested that the CpxR/CpxA system may indirectly influence the antibiotic sensitivity of the PhoP/PhoQ and PmrA/PmrB systems by regulating the activity levels of MgrB (Wang et al., 2020).

### Mildly acidic pH

pH regulates crucial biological processes such as genes expression, energy generation, and various enzyme functions. Many bacteria, including *E. coli*, *S. enterica*, *P. aeruginosa*, and *Edwardsiella*, have evolved distinct acid resistance mechanisms (Du et al., 2021; Mallick and Das, 2023). In addition to combating AMPs pressure within phagosomes, bacteria also face the challenge of phagosomal acidification that needs to be overcome (Di et al., 2017). The regulatory response to acid stress is achieved through the coordinated action of various regulators and regulatory systems (Krin et al., 2010). Two-component systems (TCS), such as PhoP/PhoQ, PmrA/PmrB, EvgS/EvgA, SsrA/SsrB, RstA/RstB, and CpxA/CpxR system consist of multiple components that enable bacteria to sense acidic environments and respond to acid stress (Perez and Groisman, 2007; Lin et al., 2017; Sen et al., 2017; Xu et al., 2020; Li and Yao, 2022; Wan et al., 2024). Deletion of *phoPQ* in *E. coli* leads to reduced expression levels of various acid-regulated proteins, highlighting the importance of PhoPQ under mildly acidic conditions. Multiple studies indicate that PhoPQ is an effective bacterial defense mechanism against phagosomal killing. PhoPQ directly regulates the lipid A deacylase PagL and the putative dehydrogenase/reductase (SDR) HlyF (Elhenawy et al., 2016; Martynowycz et al., 2019). The upregulation of their transcription levels mediates LPS modification and reshaping of lipid structures (formation of OMV) (Martynowycz et al., 2019). The periplasmic sensor PhoQ detects acidic stimuli and initiates a positive phosphorylation response, thereby activating the transcription of PhoP and its downstream acid resistance-related genes (Tu et al., 2006). Moreover, under acidic environmental conditions, PhoQ does not affect its response to other environmental stimuli (Prost et al., 2007; Choi and Groisman, 2017; Roggiani et al., 2017). For example, within macrophage phagosomes, the PhoP/PhoQ system in bacteria can simultaneously sense stimuli from cationic AMPs and mildly acidic environmental conditions. This capability reduces the damage caused by these stimuli to the outer membrane and maintains normal physiological functions of the bacteria (Han et al., 2023). PhoQ simultaneously sensing both signals rather than individually responding to one of them results in a significant increase in the abundance of PhoP and its downstream target genes (Prost et al., 2007). Under conditions of high or low concentrations of Mg^2+^, low pH can still be sensed by PhoQ (Prost et al., 2007). Be more specific about the concentration of Mg^2+^ and low pH. Research has reported that in *S. enterica*, low pH conditions lead to an increase in PhoP-P levels, which indirectly promotes transcription of *pmrD* (Perez and Groisman, 2007). This mediation enhances LPS modification effectiveness under acidic conditions, thereby strengthening bacterial resistance (Perez and Groisman, 2007). It can be seen that low pH can cooperate with other stimulating conditions to activate PhoQ, but currently, there is limited research on this aspect.

In *S. enterica* under weakly acidic conditions (pH 4.9), the UgtL protein is essential for activation of the PhoP/PhoQ system (Choi and Groisman, 2017). UgtL interacts with PhoQ, enhancing its autophosphorylation and increasing the intracellular abundance of phosphorylated PhoP (Choi and Groisman, 2017). This leads to the activation of downstream gene transcription by PhoP. However, under other stimulating conditions, the effect of UgtL on PhoQ is not significant (Choi and Groisman, 2017). Recent studies have shown that UgtS, a novel inner membrane protein homologous to UgtL, is upregulated at the transcriptional level by PhoP phosphorylation (Salvail et al., 2022). It acts as an antagonist to UgtL within macrophages of *S. Typhimurium* (Salvail et al., 2022). Following activation of the SsrB/SsrA system in response to weak acid conditions, further enhancement of *ugtL* gene expression can increase PhoP phosphorylation (Choi and Groisman, 2020a). Conversely, PhoP phosphorylation can also increase transcription of the ssrB gene (Choi and Groisman, 2020a). The PhoP/PhoQ and SsrB/SsrA systems play crucial regulatory roles in controlling genes within the (*S. Typhimurium*) pathogenicity island.

**Figure 4:**
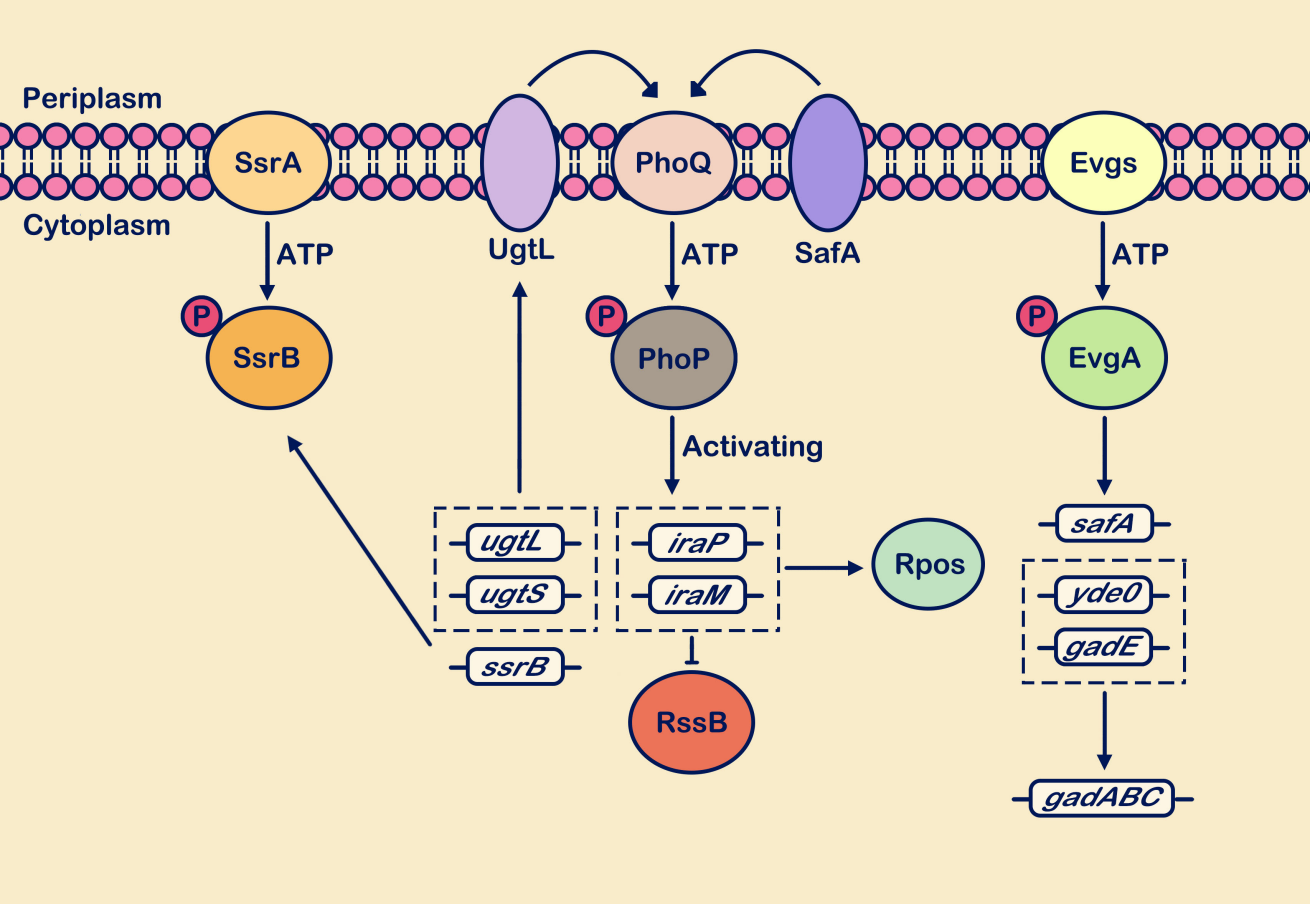
Weakly acidic pH activates multiple TCSs. Sensing the weakly acidic stimulus, the PhoP/PhoQ, EvgS/EvgA, and SsrA/SsrB systems respond positively. The PhoP/PhoQ system cascades to enhance transcription levels of genes including *ugtL*, *ugtS*, *iraP*, *iraM*, and *ssrB*. Upregulation of *ssrB* gene expression enhances the SsrA/SsrB system’s response to weakly acidic stimuli. Activation of *ugtL* and *ugtS* genes strengthens the interaction of the membrane protein UgtL with PhoQ. Upregulation of *iraP* and *iraM* gene transcription interferes with RssB protein degradation of RpoS. Within the EvgS/EvgA system cascade, transcriptional expression of acid resistance genes *gadABC* is promoted, along with upregulation of the *safA* gene. This gene interacts with PhoQ and positively regulates PhoQ. The circle with “P” represents a phosphate group.

Additionally, the TCS EvgS/EvgA system is also a major player in acid resistance, activating the expression of numerous acid-resistant genes (Dan et al., 2021; Zeng et al., 2021). It primarily branches into two pathways: EvgSA-YdeO and EvgSA-SafA (Zeng et al., 2021). YdeO is a critical component of glutamate-dependent acid resistance AR2, whose transcriptional upregulation activates the expression of the *gadE* gene, mediating the upregulation of AR2 effector genes (*gadABC*) (Roggiani et al., 2017). The membrane protein SafA acts as a connector between the EvgS/EvgA system and the PhoP/PhoQ system (Schellhorn, 2020). SafA and UgtL are both short membrane proteins (65 and 132 residues, respectively) that interact with PhoQ to facilitate network regulation of PhoP/PhoQ (Choi and Groisman, 2017; Yoshitani et al., 2019). However, they lack sequence similarity between each other and independently exert their functions (Choi and Groisman, 2017; Yoshitani et al., 2019). Through binding with anti-adaptor proteins, RssB reduces its interaction with RpoS, thereby mediating the upregulation of RpoS expression levels and ultimately promoting the transcriptional upregulation of *gadE* (Xu et al., 2019). As mentioned above, the MgrB protein acts as a feedback inhibitor in the PhoP/PhoQ system. The expression of the *mgrB* gene may be associated with acid resistance, as its deletion can increase the transcription levels of *iraM*, thereby promoting the activation of the acid resistance gene *gadE* (Yuan et al., 2017; Xu et al., 2019). **Figure 4** depicts a brief description of the PhoP/PhoQ system responding to acidic pH stimulation.

### Periplasmic redox state

Bacteria encounter oxidative stress responses induced by reactive oxygen species (ROS) during both natural environments and host infection (Hillion and Antelmann, 2015). Bacteria have evolved complex oxidative stress regulatory networks (Hillion and Antelmann, 2015). Before oxidative repair can occur, there is a need for oxidative redox sensors to transmit oxidative information directly or indirectly between them, thereby further regulating the expression of relevant proteins (Hillion and Antelmann, 2015). DsbA, a member of the Dsb family of oxidoreductases involved in disulfide bond formation, is commonly found in the bacterial inner membrane and periplasmic space (Eckels et al., 2021). Its transcriptional regulation depends on the PhoP/PhoQ system cascade, and it plays a crucial role in disulfide bond synthesis (Cardenal-Muñoz and Ramos-Morales, 2013; Choi and Groisman, 2016; Eckels et al., 2021). DsbA acts as a potent oxidase involved in disulfide bond formation, while DsbB re-oxidizes DsbA to form new disulfide bonds (Kadokura and Beckwith, 2014; Santos-Martin et al., 2021). The DsbA-DsbB pathway functions as a redox cycle, continuously driving proper folding and function of substrate proteins in the bacterial envelope and periplasmic space (Kadokura and Beckwith, 2014; Santos-Martin et al., 2021). Studies have shown that the transcription of *dsbA* and *mgrB* is regulated by PhoP-P (Cardenal-Muñoz and Ramos-Morales, 2013). In *E. coli*, the absence of *dsbA* directly leads to the activation of the PhoP/PhoQ system (Cardenal-Muñoz and Ramos-Morales, 2013). Meanwhile, MgrB, a membrane protein, has been shown to interact with PhoQ, exerting a dephosphorylating effect on PhoP and thereby inhibiting the activation of the PhoP/PhoQ system (Cardenal-Muñoz and Ramos-Morales, 2013). Furthermore, deletion of *mgrB* in *dsbA*-deficient strains has been found to reduce the activation of DsbA by the PhoP/PhoQ system (Cardenal-Muñoz and Ramos-Morales, 2013). However, there is currently limited research on the regulatory role of the PhoP/PhoQ system on the Dsb family of proteins.

### Hyperosmotic stress

Osmotic pressure is also among the environmental factors encountered during microbial growth (Brauer et al., 2023). High osmotic pressure caused by excessive or insufficient extracellular solutes that may have detrimental effects on bacteria (Sun et al., 2021; Brauer et al., 2023). The TCSs are important regulatory mechanisms in prokaryotic microbes for coping with osmotic stress. Components involved in osmotic regulation include OmpR/EnvZ, CpxA/CpxR, and the PhoP/PhoQ system (Yuan et al., 2017). The OmpR/EnvZ system can perceive stimuli of both low and high osmotic pressure across the outer membrane (Yuan et al., 2017). Meanwhile, the CpxAR system, responds to signals of outer membrane stress, whereas the PhoP/PhoQ system is specifically associated with high osmotic pressure (Yuan et al., 2017). During hyperosmotic stress (300 mM NaCl), cells experience water loss, growth stagnation, and an increase in the thickness of the lipid bilayer (Poolman et al., 2002). When PhoQ senses high osmolarity, there is a reorganization of lipid bilayers and transmembrane domain conformations, promoting the accumulation of osmoregulatory proteins through PhoP-P (Yuan et al., 2017). In *E. coli*, mutants lacking PhoPQ show decreased sensitivity to high osmolarity (Xu et al., 2019). PhoP-P mediates the activation of the *iraM* gene, whose increased expression prevents the binding of RssB to RpoS, thereby further enhancing PhoP/PhoQ activation (Xu et al., 2019). This process regulates the balance of intracellular osmotic pressure in bacteria (Xu et al., 2019). In *E. coli*, through individual knockout studies of *phoQ*, *phoP*, *envZ*, and *ompR*, it was found that the PhoP/PhoQ and OmpR/EnvZ systems independently perceive and respond to osmotic pressure stimuli (Xu et al., 2019).

### Other stimulus signals

Exogenous long-chain unsaturated fatty acids (LCUFAs) are transported across the bacterial outer membrane and converted into acyl-CoA derivatives, which serve as substrates for β-oxidation or membrane phospholipid synthesis (Viarengo et al., 2013; Xia et al., 2024). LCUFAs inhibit the activity of the PhoP/PhoQ system by interacting with the PhoQ periplasmic sensor, disrupting its autophosphorylation activity, and subsequently downregulating the expression of PhoP-P and its downstream target genes (Viarengo et al., 2013). However, previous studies have shown that LCUFAs do not compete for binding sites with other stimuli (Viarengo et al., 2013; Carabajal et al., 2020). In *S. Typhimurium*, the PhoP/PhoQ system is inhibited in response to LCUFAs stimulation. LCUFAs may bind to Ca^2+^, aiding in the distinction between intracellular and extracellular environmental conditions (Viarengo et al., 2013). Furthermore, as signaling molecules, LCUFAs play a regulatory role in coordinating bacterial virulence expression (Xia et al., 2024). For instance, in *S. enterica*, their presence can interact with the transcription regulators HilC/HilD, leading to the expression of the type III secretion system (Xia et al., 2024).

Lysine acetylation is a typical post-translational modification in bacteria that can regulate various cellular functions (Weinert et al., 2013). Acetylation utilizes acetyl coenzyme A as a cofactor, transferring acetyl groups via acetyltransferases (Weinert et al., 2013). During aerobic microbial growth, acetate is secreted as part of metabolic processes. Acetate can be converted into acetyl coenzyme A, mediating the occurrence of PhoP acetylation During aerobic microbial growth, acetate is secreted as part of metabolic processes. Acetate can be converted into acetyl coenzyme A, mediating the occurrence of PhoP acetylation (Ren et al., 2019). Research indicates that acetylation plays a crucial role in modulating PhoP activity, regulating changes in bacterial virulence (Ren et al., 2016). In *S. Typhimurium*, PhoP undergoes acetylation at three lysine residues (K201, K88, and K102), which inhibits the binding of PhoP-P to downstream gene promoters (Ren et al., 2016). PhoP K201 undergoes acetylation and deacetylation mediated by Pat and CobB, while PhoP K88 and PhoP K102 are acetylated by non-enzymatic acetyl phosphate (AcP) modification (Ren et al., 2019; Li et al., 2021). Acetylation of PhoP inhibits its phosphorylation (Ren et al., 2019), resulting in a 2- to 5-fold reduction in transcriptional activation of PhoP-regulated genes (Ren et al., 2019).

## The relationship between the PhoP/PhoQ system and other TCS components

The two-component systems regulate the activity of their sensors, response regulators, and subsequent proteins through feedback mechanisms to maintain the stability of the bacterial internal environment (Chen et al., 2021; Pina et al., 2021). The PhoP/PhoQ system, in response to various environmental signals, is also influenced by components of other two-componentsystems (Chen et al., 2021; Pina et al., 2021). It interacts with the PmrA/PmrB, EvgS/EvgA, RstA/RstB, SsrB/SsrA, and CpxR/CpxAsystems (Wang et al., 2020; Pina et al., 2021). They are interconnected through intermediate connectors (such as PmrD, SafA, MgrB), forming a complex regulatory network (Yoshitani et al., 2019; Yadavalli et al., 2020; Chen et al., 2021). PmrD, known as a connector protein, is a small regulatory RNA that acts as a connector and is activated by the PhoP phosphorylation mechanism (Zafer et al., 2019; Chen et al., 2021). It mediates the activation pathway of PhoP-PmrD-PmrA (Zafer et al., 2019; Chen et al., 2021). In many members of the Enterobacteriaceae family, the regulation of polymyxin resistance is primarily governed by two two-component systems: PmrA/PmrB and PhoP/PhoQ (Chen et al., 2021). These systems modulate the modification of bacterial outer membrane LPS through intricate signal transduction networks, thereby influencing bacterial resistance to polymyxins (Chen et al., 2021). Simultaneously, under conditions of magnesium deficiency, low pH environment, or strong stimulation of PhoQ, the PmrD protein also functions as a connector (Kox et al., 2000; Luo et al., 2010). Therefore, the PmrD protein plays a crucial role in the two-component signal transduction process by facilitating important information transfer. Moreover, the PhoP/PhoQ system can also act as an inhibitor of iron uptake proteins, synergizing with the PmrA/PmrB system to mount an immune response against high Fe^3+^ (Cho et al., 2006).

As stated above, SafA serves as a connector between the EvgS/EvgA and PhoP/PhoQ systems (Yoshitani et al., 2019). When *E. coli* is in a weakly acidic environment, it regulates acid resistance gene networks through the EvgS/EvgA and PhoP/PhoQ systems (Yoshitani et al., 2019). The sensor kinase EvgS detects low pH signals and activates the response regulator EvgA, subsequently initiating a cascade of gene transcription (Yamanaka et al., 2013). This pathway primarily bifurcates into two branches: one involving EvgA-YdeO-GadE, where YdeO activates GadE, leading to the regulation of various decarboxylases and providing resistance to acid stress (Yamanaka et al., 2013); the other branch includes SafA-PhoPQ-IraM-RpoS, with the membrane protein SafA acting as a connector, interacting with PhoQ to initiate a phosphorylation cascade. PhoP activates IraM to promote an increase in RpoS levels (Yamanaka et al., 2013). RpoS serves as a central regulator in response to external stresses, and its regulation of the *gadE* gene is a key strategy for combating weakly acidic environments (Chattopadhyay et al., 2015). Research has shown that *E. coli* significantly upregulates the expression levels of *gadA*, *gadB*, and *gadE* genes when exposed to low pH (pH 6) values (Hao et al., 2004).

Similar to SafA, UgtL is a membrane protein essential for PhoQ-mediated weakly acidic environmental signals, acting between the PhoP/PhoQ and SsrB/SsrA systems (Choi and Groisman, 2017; Choi and Groisman, 2020a). Under low pH conditions, UgtL interacts with the periplasmic domain of PhoQ, promoting the transcriptional levels of phosphorylated PhoP (Janssens et al., 2024). Research indicates that PhoP is a key regulator of the *S. Typhimurium.* Pathogenicity Island 2 (SPI-2) gene cluster, facilitating the cascade response of the SsrB/SsrA system (Shetty and Kenney, 2023; Janssens et al., 2024). Simultaneously, SsrB can also enhance the transcriptional expression of *phoP* and *ugtL*, thereby augmenting the network regulatory function of the PhoP/PhoQ system (Choi and Groisman, 2020a; Janssens et al., 2024). However, under low Mg^2+^ conditions (10 μM Mg^2+^), the expression of UgtL does not significantly change despite activation signals for PhoQ (Choi and Groisman, 2017; Janssens et al., 2024).

Meanwhile, there is cross-regulation of environmental stress between RstA/RstB and PhoP/PhoQ (Tran et al., 2016). Upon activation of PhoQ by low Mg^2+^ concentration (10 μM Mg^2+^) and low pH (the pH range is 5.0 to 6.5) signals, PhoP-P binds to the *rstA* promoter region, activating *rstA* gene transcription and influencing the cascade response of the RstA/RstB system (Tran et al., 2016). The RstA/RstB system specifically regulates purine metabolism, iron acquisition, biofilm formation, and tolerance to acidic environments (Tran et al., 2016). PhoP/PhoQ controls the function of RstA and mediates the transcriptional level regulation of acid-resistant genes (i.e. *asr* gene), curli-regulatory gene (i.e. *csgD* gene), and iron transport genes (i.e. *feoB* gene) (Ogasawara et al., 2007; Jeon et al., 2008; Tran et al., 2016). Environmental conditions influence the degree of cross-regulation between PhoQ/PhoP and other regulatory systems. Overall, the PhoP/PhoQ system does not solely respond to specific stimuli but is intricately interconnected with other TCS systems and regulatory networks.

## The PhoP/PhoQ system regulates the transcriptional expression of bacterial virulence factors

When activated, the PhoP/PhoQ system enables various bacteria to tolerate stresses such as low Mg^2+^ (10-50 μM Mg^2 +^), antimicrobial peptides, Mildly acidic pH (the pH range is 5.0 to 6.5), and high osmolarity. Multiple studies in the research process have shown that the PhoP/PhoQ cascade plays a crucial role in regulating virulence in various pathogenic bacteria, including *Salmonella*, *E. coli*, *Shigella*, *Yersinia*, and *P. aeruginosa* (Lin et al., 2017; Martynowycz et al., 2019; Fukuto et al., 2020; Xu et al., 2020; Cabezudo et al., 2022; Zhang et al., 2022). Deletion of the *phoP* or *phoQ* genes significantly reduces the virulence of these pathogens (Lin et al., 2017; Martynowycz et al., 2019; Fukuto et al., 2020; Xu et al., 2020; Cabezudo et al., 2022; Zhang et al., 2022). In *Shigella* strains with PhoPQ deletion, a reduced ability to withstand environmental stresses was observed, with the key virulence factor *icsA* being regulated by the PhoP/PhoQ system (Lin et al., 2017). SPI-1 and SPI-2 encode two type III secretion systems (T3SS), which are crucial for the pathogenicity of *S. enterica* (Jennings et al., 2017). PhoP/PhoQ mediates virulence by activating downstream target genes that modulate the expression of SPI-1 and SPI-2 (Lou et al., 2019). HilA acts as a positive regulator controlling the expression of SPI-1 genes, coordinated by the combined action of three AraC-like transcriptional activators: HilC, HilD, and RtsA (Lou et al., 2019). Studies have shown that *S. Typhimurium* lacking the *hilA* gene exhibit a phenotype equivalent to SPI-1 functionality deficiency (Lou et al., 2019). HilE is the most critical negative regulator of the *hilA* expression (Lou et al., 2019). Under conditions of low Mg^2+^ concentration (low magnesium was at 8 μM), PhoP binds to the *hilE* promoter, increasing *hilE* gene expression, which mediates inhibition of *hilA* gene expression and indirectly affects transcription of *hilD* and *rtsA* genes (Lou et al., 2019). These transcriptional changes in these genes highlight the significant role of PhoP in SPI-1 (Bijlsma and Groisman, 2005; Pérez-Morales et al., 2017). As mentioned earlier, the PhoP/PhoQ system activates and enhances the kinase activity of SsrB, concurrently boosting the transcriptional levels of its downstream gene cluster SpiCBA (Bijlsma and Groisman, 2005). Additionally, PhoPQ induces two small RNAs: MgrR and PinT (Westermann et al., 2016; Kim et al., 2019; Yeom and Groisman, 2021). The former responds to low Mg^2+^ levels by upregulating expression to influence Mg^2+^ homeostasis (Yeom and Groisman, 2021). The latter, under mildly acidic conditions, mediates the expression of SPI-1 and SPI-2 genes by regulating the transcription levels of *hilA* and *rtsA* (Kim et al., 2019). The *mgtC* gene plays a crucial role in pathogen virulence, and its transcription levels are upregulated during activation of the PhoP/PhoQ system, surpassing the expression levels of the virulence factor CigR (Yeom et al., 2018). MgtC inhibits ATP synthesis by suppressing the F1Fo ATP synthase, thereby reducing transcription of ribosomal RNA and simultaneously protecting PhoP from degradation (Yeom et al., 2017; Yeom et al., 2018). Previous studies have shown that the *S. Typhimurium* genes *cigR* and *mgtC* are located within SPI-3 and are part of the same transcriptional unit under MgtC-inducing conditions (Yeom et al., 2018). Research has shown that in *S.* Typhimurium, when exposed to low Mg^2+^ (10 μM Mg^2+^), the PhoP/PhoQ system indirectly regulates the expression of the pagM gene by affecting the transcription levels of *mgtA* and *mgtC* (Park et al., 2015). The PagM secreted protein, in turn, mediates a flagella-independent mode of motility (Park et al., 2015). This process helps the bacteria adapt to low Mg^2+^ environmental conditions by altering their mode of movement.

The H-NS nucleoid protein is a common negative regulatory protein that readily binds to AT-rich sequences, leading to silencing of associated genes (Choi and Groisman, 2020b). In *S. Typhimurium*, the SPI gene clusters exhibit higher AT content in their promoter sequences compared to ancestral genes, enhancing the pronounced negative regulatory role of H-NS, which plays a crucial role in virulence expression (Hu et al., 2019). Upon activation of the PhoP/PhoQ system, the transcription levels of downstream target genes, *ssrB* and *slyB* are upregulated. PhoP interacts with SsrB and SlyB to counteract H-NS-mediated silencing (Choi and Groisman, 2020a). Under weakly acidic conditions, the abundance of H-NS is significantly lower compared to neutral pH states (Krin et al., 2010; Choi and Groisman, 2020b). This suggests that activation of the PhoP/PhoQ system plays a crucial regulatory role in relieving H-NS-mediated gene silencing mechanisms. Previous studies have found that the PhoP/PhoQ system negatively regulates bacterial flagella (Adams et al., 2001; Janssens et al., 2024). When acid-adapted *Salmonella* (pH 5.0) is exposed to pH 3.0 conditions, the transcription level of the *fliC* gene is significantly downregulated, inhibiting flagella expression (Adams et al., 2001). This may help *Salmonella* avoid excessive activation of the host immune system (Adams et al., 2001). Overall, the PhoP/PhoQ system influences bacterial virulence systems directly or indirectly, adjusting the expression of relevant genes under different stimulus signals to maintain bacterial internal environmental stability.

## Summary and outlook

Bacteria perceive different ecological niches within the host to evade attacks from the host immune system by regulating the expression levels of relevant genes. The PhoP/PhoQ system is the most extensively studied TCS to date, and it is highly conserved across both pathogenic and non-pathogenic bacteria. The PhoP/PhoQ system senses external environmental stimuli through the dual-function membrane protein PhoQ, which, upon phosphorylation, transfers phosphate groups to the response regulator, PhoP. PhoP then regulates the abundance of downstream target genes in response to external environmental signals until the components return to stable levels upon restoration of bacterial physiological balance (Xu et al., 2019). The gene products obtained at different levels during the cascade reaction of the PhoP/PhoQ system integrate into the regulatory circuit, influencing changes in closely associated regulatory proteins and phenotype modifications (Choi and Groisman, 2020a). As mentioned earlier, the cascade reaction of PhoP/PhoQ reduces the modification of LPS, decreasing the overall negative charge of the bacterial membrane. This enhances bacterial tolerance to extreme environments, including increased resistance to antibiotics, stabilizing cytoplasmic pH, and releasing Mg^2+^ ions, among other effects. The interaction of the PhoP/PhoQ system with other TCS systems forms a complex regulatory network, collectively controlling bacterial cellular activities and virulence. Theoretically, this strategy establishes resilience and infection capabilities that can harm host cells without negative effects on the bacteria.

In summary, the PhoP/PhoQ system regulates the physiological, biochemical, antibiotic resistance, and virulence characteristics of bacteria across various environments. Moreover, it exhibits intricate synergistic interactions with other components of the TCS regulatory network. Although the PhoP/PhoQ system has received considerable attention in the past, research on its signal transduction mechanisms has primarily focused on enteric pathogens, with studies in other bacteria being relatively scarce. Studying the specific mechanisms of action of the PhoP/PhoQ system in other pathogenic or non-pathogenic bacteria, as well as its interactions with other regulatory networks, contributes to the development of effective antimicrobial therapies and mitigates the negative impacts of antibiotic use.
